# Organoids of the Female Reproductive Tract: Innovative Tools to Study Desired to Unwelcome Processes

**DOI:** 10.3389/fcell.2021.661472

**Published:** 2021-04-20

**Authors:** Ruben Heremans, Ziga Jan, Dirk Timmerman, Hugo Vankelecom

**Affiliations:** ^1^Laboratory of Tissue Plasticity in Health and Disease, Cluster of Stem Cell and Developmental Biology, Department of Development and Regeneration, KU Leuven (University of Leuven), Leuven, Belgium; ^2^Cluster Woman and Child, Department of Development and Regeneration, KU Leuven, Leuven, Belgium; ^3^Department of Obstetrics and Gynecology, University Hospitals, KU Leuven, Leuven, Belgium; ^4^Department of Gynecology, Klinikum Klagenfurt, Klagenfurt, Austria

**Keywords:** organoids, gynecology, reproduction, cancer modeling, women’s health

## Abstract

The pelviperineal organs of the female reproductive tract form an essential cornerstone of human procreation. The system comprises the ectodermal external genitalia, the Müllerian upper-vaginal, cervical, endometrial and oviductal derivatives, and the endodermal ovaries. Each of these organs presents with a unique course of biological development as well as of malignant degeneration. For many decades, various preclinical *in vitro* models have been employed to study female reproductive organ (patho-)biology, however, facing important shortcomings of limited expandability, loss of representativeness and inadequate translatability to the clinic. The recent emergence of 3D organoid models has propelled the field forward by generating powerful research tools that *in vitro* replicate healthy as well as diseased human tissues and are amenable to state-of-the-art experimental interventions. Here, we in detail review organoid modeling of the different female reproductive organs from healthy and tumorigenic backgrounds, and project perspectives for both scientists and clinicians.

## Introduction

**FIGURE 1:**
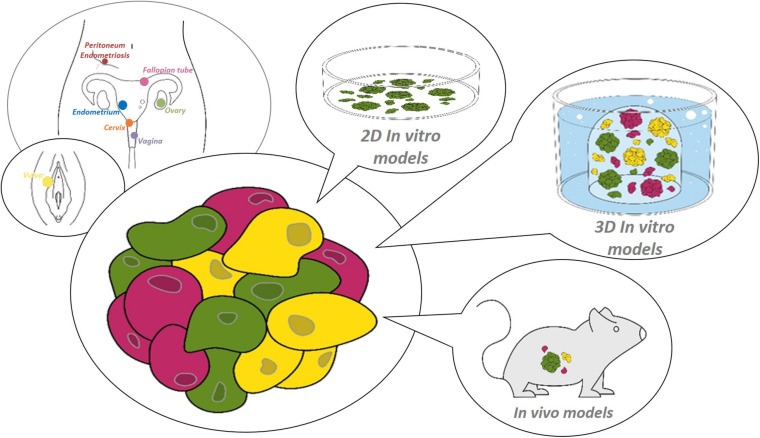
Research models for the female reproductive tract. Starting from healthy or diseased tissue from the site of interest within the female reproductive tract (from distal to proximal: vulva, vagina, cervix, endometrium, fallopian tube, ovary and peritoneum/endometriosis), (patho-)physiology can be studied using various preclinical 2D or 3D *in vitro*, or *in vivo* models.

**FIGURE 2:**
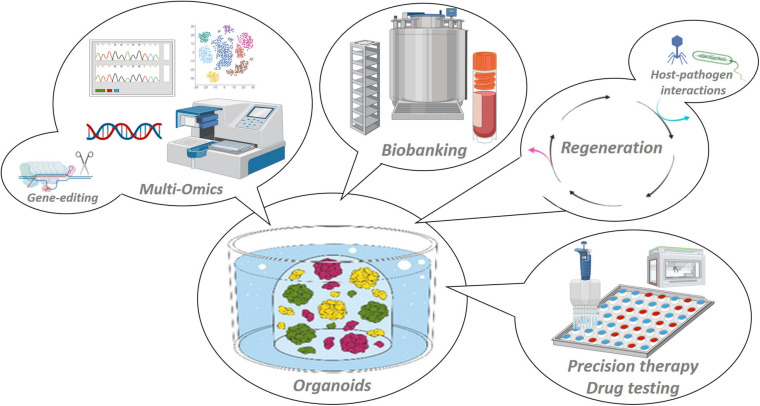
Applications of organoid model systems. Organoids are stable 3D *in vitro* representations of the tissue of origin that adequately recapitulate tissue (patho-)biology, and are amenable to manifold basic and (pre-)clinical research applications such as multi-omic scrutiny and gene-editing, host–pathogen interaction mapping and regenerative medicine, biobanking and high-throughput drug testing toward patient-tailored treatments.

The female reproductive system serves a unique purpose as it harbors the beginning of life, but, conversely, also risks to bring about the very end of it. The embryonic etiology of these closely related tissues are threefold (Brauer, 2009; Mutter and Robboy, 2014). The vulva and lower third of the vagina arise from ectoderm. The tissues making up the actual female reproductive tract (FRT), including upper two-thirds of the vagina, cervix, uterus, endometrium and fallopian tubes (FT), are mesodermal derivatives whereas the ovaries originate from the endoderm. Each of these tissues is epitomized by its unique patterns of development, proliferation and differentiation. Importantly, to serve their reproductive purpose, these tissues rely on the self-renewing capacity of their constituents (Patterson and Pru, 2013). However, it is in their efforts that allow for life to begin, by fulfilling transportation functions, supporting implantation and serving as barriers from internal and external stressors and pathogens, that its constituents risk cellular deregulation due to threatening impacts such as hormonal dysregulation, infections or auto-immune diseases, which may ultimately result in carcinogenesis (Hanahan and Weinberg, 2011; Visvader, 2011). In general, insight in tissue development, homeostasis and disease has been obtained from several research models that throughout the years have become more complex and representative, and even personalized (Schutgens and Clevers, 2020). For many years, hypothesis testing for tissues of the FRT has relied on two-dimensional (2D) models (such as cell lines) that have stood alongside short-term three-dimensional (3D) *in vitro* cell-culture (such as spheroids) and *in vivo* explant systems [such as patient-derived xenografts (PDXs)] (Adissu et al., 2007; Gargett et al., 2016; Figure 1). It took until 2009, however, for the prospect of a complete transition into use of long-term 3D *in vitro* cell-culturing methods to be envisioned. The intestine was the first of many organs to have its stem cell niche analyzed and have expansion and differentiation pathways charted by means of a self-forming and -organizing, tissue stem cell-derived culturing system called “*organoids*” (Ootani et al., 2009; Sato et al., 2009). The insights brought about by this novel technology, reliant on the use of a gel-based substitute for the local extracellular matrix (ECM) and supplementation of feeder- and serum-free stem cell niche-supporting factors, were manifold (Kleinman et al., 1986). This system was the first to show defined use of growth and regulatory factors required by the stem cell niche, thereby discarding the need for highly variable and ill-defined serum supplements (Clevers et al., 2014). Contrary to 2D cell lines, organoids proved to remain morphologically, genomically and transcriptomically stable over a long period of time and serial passaging. This novel culturing method thereby allowed for spatiotemporal tracking (i.e., how various specific cell types occupy different positions within tissues and how their positions alter through time) of developing organs, requiring only the bare minimum of starting (patient) material. Rapidly and effectively, the complex biology of the epithelial compartment of the intestine was analyzed for the entire spectrum spanning healthy to diseased conditions (Sato et al., 2009, 2011; van de Wetering et al., 2015; Hibiya et al., 2017). Although revolutionizing human research, organoids were only replicating the tissue’s epithelium. This drawback of lacking the organ’s stromal, vascular and immune cells and their interplay was readily overcome by the development of various *in vitro* co-culturing as well as *in vivo* transplant modalities (Nozaki et al., 2016; Roper et al., 2017; Dijkstra et al., 2018). Overall, more reliably than other experimental models, organoids enable a wide variety of basic, translational and clinical research prospects such as deciphering the heterogeneous make-up of tissues via multi-omic analyses, unraveling host tissue-pathogen interactions, and advancing precision and regenerative medicine using cryopreserved and biobanked organoid lines (Figure 2). Organoids are also amenable to cutting-edge experimental technologies such as CRISPR-Cas9 gene editing, and can be efficiently subjected to live imaging (Kim et al., 2021).

In this review, we aim to systematically list the efforts made in the field of organoid research for the tissues that make up the human FRT. We provide a systematic overview of the organoid models developed and the growth media that detail the niche requirements ranging from healthy to diseased states. We critically appraise their validity and scrutinize reports for investigated applications. Taken together, we aim to highlight the specific benefits of organoid technology in the setting of desired and unwelcome processes of the human FRT.

## Vulva

The vulva, that consists of labia majora, mons pubis, labia minora, clitoris and vestibulum, acts as a gatekeeping structure and serves as a first line of defense in order to protect the FRT against extracorporeal stressors. Being an ectodermal derivative, the vulva is shaped as urogenital or cloacal folds through cellular expansion of its underlying mesodermal compartment (Brauer, 2009). As in other tissues (Sato et al., 2011), this epithelium serves not only as a mechanical scaffold, but also as a source for paracrine crosstalk that shapes the specialized cellular niche, thereby anchoring and supporting resident stem cells. To date, no organoids have been derived from appendages of the human perineal or vulvar region (Table 1 and Supplementary Table 1). Insights on vulvar homeostasis and disease can, however, be inferred from organoid studies exploring skin because the vulva, forming the exterior ending of the FRT, is largely covered with skin epithelium (Lei et al., 2017; Gupta et al., 2018; Boonekamp et al., 2019; Diao et al., 2019). Lei et al. (2017) elucidated the spatiotemporal component in mouse epidermal development using organoids, thereby unveiling which transcriptional pathways are consecutively activated during each phase of skin development. Boonekamp et al. (2019) established an organoid system for long-term expansion of murine keratinocytes and were able to initiate and maintain cultures from stem cells with various gene signatures. The fact that these organoids were amenable to genetic manipulation may draw a parallel toward prospective vulvar organoids in which gene alterations may be studied as, for instance, occurring during chronic inflammation (e.g., lichen sclerosus or lichen planus) or infection (e.g., candidosis or herpes genitalis) or in driver genes involved in blistering diseases (e.g., epidermolysis bullosa or pemphigus vulgaris) and carcinogenesis (e.g., basocellular or squamous vulvar cancer). Organoids may also be particularly useful for drug screening in order to find (new) drug targets and drugs that can mitigate the undesired vulvar diseases. On the vulva like on skin, sweat glands serve as gatekeepers for bacterial colonization, waste excretion and body-temperature maintenance. These glands are therefore important elements to explore when trying to understand vulvar (microbial) homeostasis. Diao et al. (2019) generated an organoid culture system for epidermal sweat glands, which may pave the way to vulvar sweat gland-derived organoids. Gupta et al. (2018) studied epithelial–mesenchymal interactions in composite organoids obtained by co-culturing human dermal papilla spheroids, hair follicle keratinocytes and stem cells in a hydrogel-based microenvironment. Taken together, vulva-derived organoids will be powerful tools to help understanding normal epithelium biology and microbiome interaction, and specific vulvotropic diseases such as genital infections and cancers (e.g., vulvar squamous cell carcinoma), as well as to provide a potential means for tissue regeneration after debilitating surgery (e.g., radical vulvectomy).

**TABLE 1:** Main findings and applications of human female reproductive tract organoid studies.

Organoids of human …	Author, year	Main findings	Applications
VULVA	NA	NA	NA
VAGINA	NA	NA	NA
CERVIX	Chumduri et al., 2018, 2021	• Differential niche requirements for squamous and columnar cervical organoids suggest cervical homeostasis is determined by stromal Wnt signaling rather than epithelial transition. • Squamous cancers probably originate from CK5^+^, adenocarcinomas from CK7^+^CK8^+^ cells.	Characterization, biobanking
	Maru et al., 2019a	Establishment of cervical clear cell carcinoma organoids	Characterization, biobanking, xenografting, drug screening
	Maru et al., 2020	Establishment of normal and metaplastic cervical organoids from the squamocolumnar junctional zone	Characterization, biobanking
ENDOMETRIUM			
Healthy endometrium	Turco et al., 2017	• Establishment of endometrial organoids of all phases of menstrual cycle and decidual changes. • Endometrial organoids are clonogenic and bipotent.	Characterization, optimization, biobanking,
	Boretto et al., 2017	Establishment of endometrial organoids of all phases of menstrual cycle. • Human endometrial organoids express *LGR4* and *LGR5* and WNT ligands are endogenously expressed. • Endometrial organoids mimic the menstrual cycle in a dish.	Characterization, optimization, biobanking
	Haider et al., 2018	• Establishment of trophoblast organoids. • Wnt signaling promotes villous but not extravillous trophoblast formation.	Characterization, optimization, biobanking
	Turco et al., 2018	Establishment of trophoblast organoids	Characterization, optimization, biobanking
	Fitzgerald et al., 2019	• Validation of endometrial organoid model. • Deepened understanding of gene expression upon hormonal stimulation.	Characterization, biobanking
	Hennes et al., 2019	• Validation of endometrial organoid model. • Mechanosensitive ion channels (e.g., PIEZO1) are expressed in endometrial organoids.	Characterization, mechanical stimulation, patch clamping, calcium imaging, drug screening,
	Haider et al., 2019	• Validation of endometrial organoid model. • Estrogen and NOTCH signaling drive ciliogenesis.	Characterization, biobanking, drug screening
	Luddi et al., 2020	• Validation of endometrial organoid model. • Receptivity marker glycodelin A differs between healthy and endometriosis-affected endometrium.	Characterization
	Cochrane et al., 2020	• Validation of endometrial organoid model. • Differentiation of secretory and ciliated epithelial cell populations in endometrial organoids.	
	Marinić et al., 2020	Endometrial gland organoid derivation from term placentas	Characterization
Adenomyosis and endometriosis	Boretto et al., 2019	• Establishment of endometriosis organoid model. • *LGR6* is upregulated in endometriosis organoids. • Inflammatory and cancer-associated genes/traits are found in endometriosis organoids.	Characterization, optimization, biobanking, xenografting, drug screening
	Esfandiari et al., 2021	Validation of endometriosis organoid model	Characterization
Endometrial hyperplasia and cancer	Dasari et al., 2017	Verteporfin as promising therapeutic agent for endometrial cancer.	Characterization, drug screening
	Girda et al., 2017	• Establishment of endometrial cancer organoid model. • Novel STAT3 inhibitors as potent anticancer agent.	Characterization, drug screening
	Pauli et al., 2017	Combination of buparlisib with olaparib as optimal treatments in endometrial organoid and PDX models.	Characterization, biobanking, drug screening, xenografting
	Boretto et al., 2019	• Establishment of endometrial cancer (-predisposed) organoid models. • Significant differences compared to healthy endometrium in PIEZO1 and transient receptor potential channels.	Characterization, optimization, biobanking, xenografting, drug screening, calcium imaging, patch clamping
	Maru et al., 2019b	Establishment of endometrial cancer organoid model.	Characterization, optimization, biobanking, drug screening
FALLOPIAN TUBES	Kessler et al., 2015	• Establishment of healthy fallopian tube organoid model. • Fallopian tube stemness is Wnt- and NOTCH-dependent.	Characterization, optimization, biobanking
	Kessler et al., 2019	*Chlamydia* infection can be mimicked in oviductal organoids and increases DNA hypermethylation and stemness.	Characterization
	Kopper et al., 2019	• Establishment of healthy fallopian tube organoid model from BRCA germline mutation carriers. • Strong Wnt dependency in fallopian tube organoids.	Characterization, optimization, biobanking, drug screening, gene-editing
	de Witte et al., 2020	• Fallopian/ovarian cancer organoid response matches patient’s clinical response. • Intra- as well as inter-patient drug response heterogeneity.	Characterization, optimization, biobanking, drug screening, clinical correlation
	Hoffmann et al., 2020	• Triple knock-down oviductal organoids show ovarian cancer traits. • Medium optimized for ovarian cancer organoids promotes stemness in modified oviductal organoids. • Modified organoids thrive in Wnt-free environment.	Characterization, optimization, biobanking, drug screening, clinical correlation
	Rose et al., 2020	• Fimbrial ends of the oviducts possess the highest organoid-forming capacity. • Aldehyde dehydrogenase-positive cells replicate with higher frequency and form larger structures.	Characterization,
OVARIES	Hill et al., 2018	• Establishment of short-term ovarian cancer organoids. • Functional assays of homologous repair deficiency outperform isolated assessment of mutational profiles.	Characterization, drug screening
	Kopper et al., 2019	• Establishment of (predisposed) healthy and diseased ovarian organoid model. • Importance of heregulin-β1 (neuregulin-1) and low WNT environment.	Characterization, optimization, biobanking, drug screening, xenografting
	Maru et al., 2019b	Establishment of ovarian cancer organoid model	Characterization, optimization, biobanking, drug screening
	de Witte et al., 2020	• Fallopian/ovarian cancer organoid response matches patient’s clinical response. • Intra- as well as inter-patient drug response heterogeneity.	Characterization, optimization, biobanking, drug screening, clinical correlation
	Hoffmann et al., 2020	Ovarian cancer organoids require low-Wnt environment	Characterization, optimization, biobanking, drug screening, clinical correlation
	Maenhoudt et al., 2020	• Establishment of ovarian cancer organoid model. • Importance of heregulin-β1 (neuregulin-1).	Characterization, optimization, biobanking, drug screening
	Chen et al., 2020	Establishment of short-term organoids/spheroids model from malignant effusion fluids	Characterization, biobanking, drug screening
	Nanki et al., 2020	Establishment of ovarian cancer organoid model	Characterization, drug screening
	Zhang et al., 2020	Establishment of ovarian cancer organoid model	Characterization, drug screening

## Vagina

Like for the vulva, most of our current understanding on vaginal development and regeneration is construed from 2D primary cell culture and animal experiments (Bulmer, 1957; Mutter and Robboy, 2014). Mouse studies have been essential in conveying the importance of a transformative interplay between the epithelium and its underlying stroma, revealing that the stroma eventually induces cytodifferentiation of pseudostratified columnar to squamous epithelium and shapes the morphology of the overlying epithelium (Cunha, 1976; Robboy et al., 1982; Iguchi et al., 1983; Tsai and Bern, 1991; Miyagawa and Iguchi, 2015). Several mouse-derived 2D cell culture and *in vivo* studies later underscored the role of hormone receptor genes and hinted on the possible contribution of the Wnt/β-catenin pathway to vaginal proliferation and differentiation (Iguchi et al., 1983; Tsai and Bern, 1991; Nakamura et al., 2012; Miyagawa and Iguchi, 2015; Mehta et al., 2016; Li et al., 2018). Only recently, the pivotal function of the latter pathway in vaginal epithelium became elegantly clear. After discovering new bona fide markers of different subpopulations in mouse vaginal epithelium, including potential stem cell populations, Ali et al. succeeded in establishing an expandable, genomically stable 3D organoid culturing system (Ali et al., 2020). After embedding single-cell suspensions in basement membrane extract, the sole requirements appeared to be the addition of epidermal growth factor (EGF), transforming growth factor β receptor (TGF-βR) kinase inhibitor, and Rho-associated protein kinase (ROCK) inhibitor (ROCKi). EGF and TGF-βR kinase inhibitor were important throughout the whole culturing process and served as (stem cell) mitogens, whereas ROCKi was used mainly during passaging and seeding steps to prevent anoikis of the single cells. The importance of Wnt and BMP signaling in maintenance of the stem cell niche was accentuated by the positive correlation between organoid-forming capacity and concentrations of supplemented Wnt3a and R-Spondin-3 (RSPO3), both Wnt pathway activators, and of Noggin, a BMP inhibitor. Inversely, inhibiting Wnt *O*-acyltransferase Porcupine (PORCN) activity, needed for Wnt ligand maturation and secretion, via IWP-2 halted organoid growth and multiplication. Pulse labeling of cells expressing Wnt target and regulator axis inhibition protein 2 (AXIN2) in doxycycline-inducible AXIN2^rtTA^/tetO^Cre^/lacZ^fl/+^ mice led to understand that these cells give rise to all other epithelial cell lineages of vaginal epithelium in mice. Unfortunately, to the best of our knowledge, no data on vaginal epithelium stem cells are available in humans (Table 1 and Supplementary Table 1). Considering that vaginal epithelium, together with the vulva, represents the first line of defense against pathogenic colonization or infection of the reproductive organs, human vaginal organoids would allow us to gather better understanding of how these human cells interact with micro-organisms on a (sub-)cellular level. In addition, by co-culturing organoids with associated stroma, a more thorough comprehension could be obtained of the signaling cascade that causes and maintains vaginal atrophy. If eventually applied in a system that also encompasses the immune and vascular system, a more purposeful narrative could be written for pathogenesis and drug discovery in vaginal cancers. Owing to their rarity and proximity of the vulva and cervix, vaginal cancers are either treated as cervical or vulvar entities and not a single treatment algorithm is set out to deal with vaginal cancers focusing on their intrinsic characteristics. Therefore, a model that allows understanding, expanding and biobanking these rare cancers would be invaluable to gynecological cancer research (de Martel et al., 2017). Once more, organoid technology may prove here to bridge the gap between bench and bedside.

## Cervix

**FIGURE 3:**
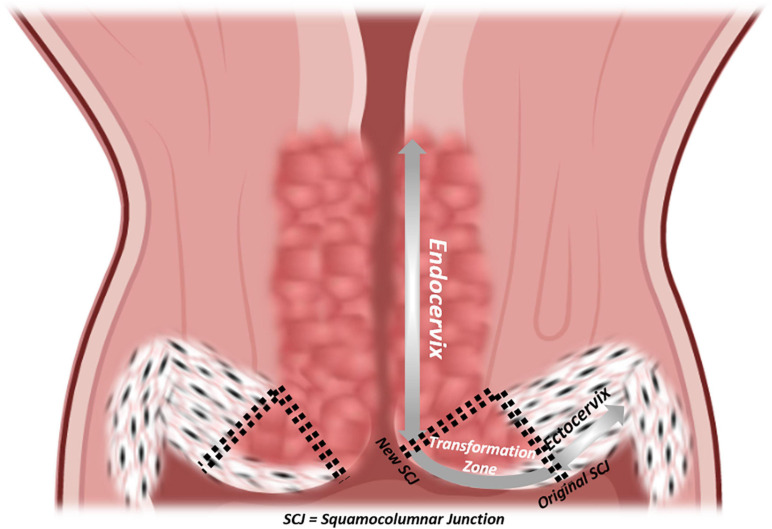
Epithelial histology of the cervix. Coronal section of the cervix with a detailed visualization of epithelia, from vagina to isthmus: ectocervix, original squamocolumnar junction (SCJ), new SCJ, endocervix.

The uterine cervix is the final frontier between a stressor-laden, entropic external environment and the well-organized, homeostatic internal conditions at the locus of implantation. Yet, more than any other compartment of the FRT, the focus of cervical research lies not in its impact on fertility, but in its risk of oncogenic transformation. It is still the most prevalent gynecological cancer worldwide. It is infection-driven, most notably with oncogenic strains of human papillomavirus (HPV) (zur Hausen et al., 1974; de Sanjose et al., 2010; de Martel et al., 2017; Bray et al., 2018). However, not all tissue-resident cells are equally prone to malignant transformation. The cervix initially comprises two distinct native epithelia with a dynamic interface that give rise to an area consisting of a third, transformative epithelium that displays traits of both precursors (Figure 3). The ectocervix is marked by a stratified, non-keratinizing epithelium similar to that of the vagina. The endocervix is an evident extension of the endometrium consisting of a single line of columnar, mucus-secreting cells, sparsely interspersed with ciliated cells. Starting from the original squamocolumnar junction (SCJ) and due to the acidity of the vaginal compared to the endocervical environment, transformative pathways are activated in bipotent progenitor, so-called “reserve” cells, subjacent to columnar cells to replace and replenish the exposed surface with metaplastic squamous epithelium (Herfs et al., 2013; Malpica, 2014). Even though carcinomas arising from both epithelia display only minute differences in clinical risk factors and majority of dedicated HPV types, much is still to be elucidated on how they pathogenetically diverge (Berrington de González et al., 2004; Bray et al., 2005; de Sanjose et al., 2010; Patterson and Pru, 2013; Deng et al., 2018; Stewart et al., 2019). It has been postulated that cytokeratin (CK-) 7-positive stem cells residing at the SCJ, may represent a cell population in which cervical carcinogenesis originates (Herfs et al., 2012, 2013). Current understanding of cervical cancer cells has been acquired owing to the efforts of Gey et al. (1952) who established the renowned “HeLa” cancer cell line from an aggressive clone of cervical adenocarcinoma, named after its patient donor Henrietta Lacks. In general, the establishment of cervical cancer cell lines provided important stepping stones toward more insight into molecular and genetic cancer pathways, but these 2D cell-line models suffer from major shortcomings. First, only highly aggressive tumors can be readily established in cell lines (Gartler, 1968; Sandberg and Ernberg, 2005). Second, the tumor niche is only poorly recapitulated, using undefined supplements such as chicken plasma, bovine embryo extract and human placental cord serum, or more in general, serum (Gartler, 1968; Sandberg and Ernberg, 2005). Third, clonal selection (with loss of tumor original heterogeneous composition), genetic drift and contamination by other cell lines, all severely compromise their use as representative cancer research models (Gartler, 1968; Sandberg and Ernberg, 2005). Since cervical cancer is a predominantly infection-mediated disease, scientists turned to HPV-transfected (immortalized) keratinocytes and direct (epi-)genetic analysis on patient samples to study its pathogenesis, thereby unveiling roles for pathways impacting apoptosis and cell-cycle inhibition (Dyson et al., 1989; Scheffner et al., 1990; Tsuda et al., 2003; Cancer Genome Atlas Research Network, 2017). The importance of three-dimensionality in studying HPV life cycle was underpinned by the application of a raft culture method encompassing immortalized human foreskin keratinocytes on a dermal-equivalent support at air–liquid interface, allowing to study short-term events in the non-productive stages of HPV transmission, as well as impact of viral persistence and replication in the process of tissue stratification and differentiation (Flores et al., 1999). Co-culturing with immune cells allowed for a still better approximation of the genital mucosal microenvironment (Delvenne et al., 2001). A further advanced 3D set-up, using ECM-bound virions to infect primary foreskin keratinocytes and subsequently culturing these ensembles as rafts, recapitulated the earliest events of HPV infection as well as viral persistence, disease progression and viral invasion, thereby providing invaluable insights in the first steps of viral infection and unveiling which viral transcripts are sequentially activated (Bienkowska-Haba et al., 2018). However, a specific fibroblast feeder cells providing stromal signals were used in these models which may be overcome by applying 3D organoid technology (Table 1 and Supplementary Table 1). Recently, Maru et al. (2020) succeeded in establishing cervical organoids from a limited number of patient-derived biopsies in a medium supplemented with RSPO1, Noggin, EGF, ROCKi and the Notch ligand Jagged-1. The organoid cells expressed validated SCJ markers more robustly than classic cell lines and displayed both differentiated endo- and ectocervical cell types. Chumduri et al. (2018, 2021) generated endocervical-like, long-term expandable organoids from ecto- and endocervical patient samples, dependent on the presence of Wnt agonists (RSPO1 and WNT3A). They showed differentiation potential toward an ectocervical phenotype by activating the cAMP pathway and hinted toward different originating cells (i.e., squamous cancers from CK7^+^ and adenocarcinomas from CK7^+^CK8^+^ cells). Maru et al. (2019a, b) were also able to establish organoids from a cervical clear cell carcinoma using their previously published culture conditions for other gynecological cancers. This organoid line, as well as endometrial and ovarian cancer organoids, were subjected to tailored drug therapy and grown as xenografts in the dorsal skin of immunocompromised nude mice (Maru et al., 2019a, b). However, although orthotopic xenograft models have previously been advocated as promising tools to model cervical cancer (Hoffmann et al., 2010), there are main limitations including lack of translatability because of species differences in stromal and immune cell interactions (in the PDX model, originating from mouse), the take rate mostly limited to aggressive subtypes, and the highly time- and animal-consuming aspects. Organoid technology may overcome some of these hurdles, as an impetus to still more advanced co-culture systems including immune cells. For instance, much remains to be learned about the effect of the genital mucosal microenvironment on virus-specific effector and suppressor immune responses and their impact on lesion pathogenesis.

## Endometrium

### Healthy Endometrium

**FIGURE 4:**
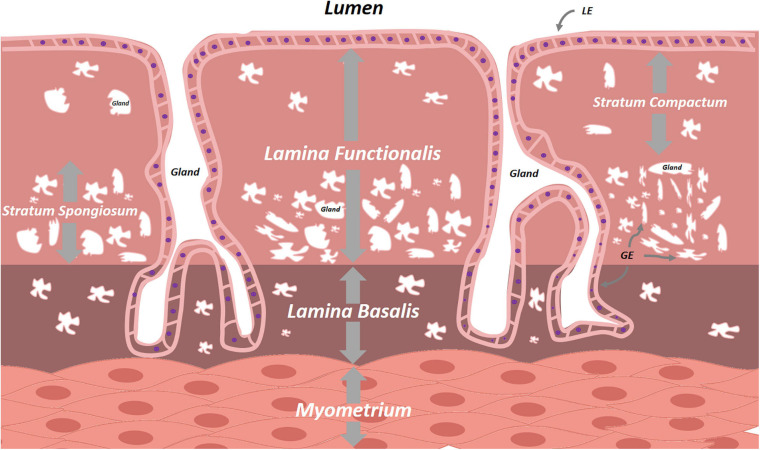
Schematic overview of the endometrium. The cyclically replenished *lamina functionalis* divides into the superficial *stratum compactum* and the deeper *stratum spongiosum*. The persistent *lamina basalis* lies between the *lamina functionalis* and the uterine myometrium. Both lamina contain epithelial cells interspersed with varying densities of stromal cells. LE, luminal epithelium, GE, glandular epithelium.

The multilayered inner lining of the uterus plays a pivotal role in human reproduction. Its make-up is tightly regulated by the hypothalamic-pituitary-gonadal axis and, in order to serve its primordial purposes of embryonic implantation and nourishment, it undergoes monthly reiterative cycles of proliferation, differentiation and menstrual shedding (Yin and Ma, 2005). This mucosal lining lies in continuity with that of the endocervix at the proximal side and FT at the distal end, and consists of glands, stroma, blood vessels and immune cells. Histologically subdivided, the endometrium consists of the - durably present - *lamina basalis* deeply and adjacent to the myometrium, and the - menstrually shed - *lamina functionalis* more superficially (Figure 4). The *lamina functionalis*, containing luminal epithelium (LE) and glandular epithelium (GE), organizes into both a deeper lying *stratum spongiosum*, marked by numerous glands and ensuing loose stromal organization, and a superficial, less glandular and thereby stromally dense, *stratum compactum*. Whether the monthly regeneration of the endometrium is driven by endometrial stem cells, remains unclear and actively debated. Several epithelial and stromal stem cell candidates have been proposed, including long-term label-retaining cells, endometrial side population cells, perivascular CD146+, platelet-derived growth factor receptor-β (PDGFR-β+) and sushi domain containing-2 (SUSD2+) cells, and AXIN2+ cells, the latter identified by lineage tracing in mice and found responsible for tissue regeneration (Cunha, 1976; Robboy et al., 1982; Gray et al., 2001; Patterson and Pru, 2013; Gargett et al., 2016; Tempest et al., 2018; Syed et al., 2020). In addition, contribution of bone marrow-derived (endometrial progenitor) cells has also been suggested during the cyclic regeneration. Many questions and controversies remain regarding potential (hierarchical) relationship between the various stem cell candidates, and several of these findings in mice have not been translated into human. Apart from comparative ungulate, rodent or primate studies, understanding of regeneration-involved signaling pathways between cells, or between cells and ECM, sprouted from direct immunohistochemical (IHC) time-point analyses in human tissue (Snijders et al., 1992; Thiet et al., 1994; Jones et al., 1998; Aflatoonian et al., 2007). Also here, 3D models, cultured in basement membrane mimics, would be highly interesting to decipher these cellular processes and crosstalks. Iguchi et al. (1985) were able to dissociate luminal mouse endometrium from its fibromuscular stromal surroundings and seed these cells on collagen gel matrices in serum-free conditions. Cultures were short-lived, but the endometrial cells demonstrated apicobasal polarity and were characterized by spherical outgrowth and sheet- and/or duct-like extrusions, reminiscent of adenogenesis *in vivo*. Rinehart et al. (1988) seeded 3D endometrial glands in 50% Matrigel on Matrigel-coated plates. However, the 3D structures spread out into 2D monolayer colonies with columnar aspect, apicobasal polarity and preserved intercellular connections, but were not deeply characterized. Moreover, the medium, albeit serum-free, was not entirely chemically defined as 10% was made up of conditioned medium of the RL95-2 endometrial carcinoma cell line. Efforts by other groups showed persistent requirement of low amounts of serum, were hampered by the inability of long-term maintenance or expansion, and did not adequately recapitulate the endometrium as observed *in vivo* (Bentin-Ley et al., 1994; Bläuer et al., 2005, 2008). Only recently, human endometrium-derived organoids were successfully derived, using a defined culture medium (Boretto et al., 2017; Turco et al., 2017; Haider et al., 2019) (Table 1 and Supplementary Table 2). Activation of Wnt/β-catenin signaling with RSPO1 (or CHIR99021, an inhibitor of β-catenin degradation) proved indispensable. This is in line with the proposed role of Wnt in uterus development and adenogenesis (uterine gland formation), recently supported by *in vivo* lineage tracing of AXIN2+ cells, assigning these cells as plausible stem cell candidates in mouse uterus (Syed et al., 2020).

Different from mouse endometrial organoids, development and culture of human organoids did not require exogenous WNT3A. Inhibition of BMP (Noggin) and TGF-β/Alk (A83-01) pathways was indispensable, plausibly quenching differentiation of the organoid-driving stem cells (Boretto et al., 2017). Cultures furthermore benefited from the mitogens EGF and fibroblast growth factor 10 (FGF10), the endometrium-proliferative hormone 17β-estradiol (E2), insulin transduction activation (insulin-transferrin-selenium, ITS), and inhibition of p38 MAPK (SB202190 [p38i]), of reactive oxygen species (*N*-Acetyl-L-Cysteine [NAC]), of ROCK (Y-27632 [ROCKi]) and of sirtuin (nicotinamide [NAM]). By exposure to a specific hormone treatment protocol encompassing E2 and progesterone (P4), organoids mimicked the full menstrual cycle as well as incipient decidualization (Table 1 and Supplementary Table 3). The robustness of the endometrial organoid platform has been validated by IHC, electron microscopy, array Comparative Genomic Hybridization (aCGH) and transcriptome analysis and, in addition, ion channel functionality and ciliogenesis was demonstrated using these organoid systems (Fitzgerald et al., 2019; Haider et al., 2019; Hennes et al., 2019; Cochrane et al., 2020; Bui et al., 2020; Luddi et al., 2020; Syed et al., 2020). Two groups achieved to derive organoids from trophoblasts, offering the appealing possibility to study trophoblast-endometrium crosstalk *in vitro* (Haider et al., 2018; Turco et al., 2018). Organoid medium of both studies only showed minimal differences, highlighting the importance of EGF, Wnt activation (RSPO, CHIR99021, and prostaglandin E2 [PGE2]) and inhibition of TGF-β signaling (A83-01) (Haider et al., 2018; Turco et al., 2018; Table 1 and Supplementary Table 3). Interestingly, Noggin was not required, but Haider et al. (2018) retained it to limit differentiation. With only few medium alterations compared to trophoblast organoid culturing, Marinić et al. (2020) were able to derive endometrial gland organoids from term placentas which showed proper hormone responsiveness and molecular patterns distinct from the endometrial stromal cells, initially also present in the term placenta samples.

Taken together, these recent realizations have provided an interesting new port of entry in pregnancy research. The organoid models may generate unprecedented insight in the signal transduction cascade at both maternal and fetal side during embryo apposition, implantation and outgrowth, may impart in-depth understanding of the pathways leading to fetal growth restriction and pre-eclampsia, and may lead us to a better apprehension of genetic placental aberrations found at chorionic villus sampling (such as confined placental mosaicisms). Also, the organoid platform may provide insight in developmental biology of iatrogenically arrested pregnancies nesting in cesarean scar tissue or uterine niches, and shed light on unsolved questions in infertility research such as non-receptive endometria or recurrent implantation failure.

### Adenomyosis and Endometriosis

Adenomyosis and endometriosis have been put forward as two distinct entities within the same continuum. Both conditions essentially display benign endometrial epithelium and stroma outside the uterine cavity, but affect different patient groups and are thought to arise through different pathogenic mechanisms. Adenomyosis boils down to the presence of ectopic endometrial tissue within the uterine wall that is fully confined within hypertrophic myometrium, supposedly owing to invagination (Frankl, 1925; Emge, 1962; Leyendecker et al., 2009). In endometriosis, endometrial tissue and cells are believed to translocate toward the peritoneal cavity by means of retrograde menstrual flow, after which they generate ectopic deposits on peritoneum and/or internal organs (Sampson, 1921, 1927; Leyendecker et al., 2009). For both diseases, however, alternative explanations - among which metaplasia is the most notable -, have been considered to remedy few remaining clinical shortcomings of the translocation theories (Gruenwald, 1942; Vannuccini et al., 2017; García-Solares et al., 2018; Koninckx et al., 2019). Valuable insights on mechanisms underlying pathogenesis and progression of both diseases have been obtained from non-human primate studies given that endometriosis naturally occurs only in this species with histopathological features consistent with the human disease (D’Hooghe et al., 1991, 1992; Fazleabas, 2010; Donnez et al., 2013). Although providing longitudinal insights in important primary endpoints such as fertility and pain-related behavior, this research approach suffers from multiple limitations including strong ethical concerns, labor-intensity and costliness, and still faces species-specific translatability restraints. Again, a myriad of mouse and *in vitro* cell culture models have been used to study endometriosis (Habiba, 2016; Greaves et al., 2017), however, encountering clear shortcomings including the inability to reproduce patient variability as well as phenotypic heterogeneity between different stages and types of endometriosis, and logically also species differences with endometriosis not natively occurring in mouse. The establishment by our group of robustly expandable 3D organoids from human endometriotic lesions, as well as from the (eutopic) endometrium of the same patients, provides more appropriate study models (Boretto et al., 2017, 2019; Table 1 and Supplementary Table 2). Organoid development efficiency appeared somewhat lower than from healthy endometrium, likely secondary to the harsher experimental conditions needed to dissociate the endometriotic lesion. Medium components were similar. Organoids were developed from different endometriosis types (including superficial and deep peritoneal lesions) and different severity stages. Transplantation of the endometriotic organoids into the renal capsule of immunocompromised mice, or more orthotopically by intraperitoneal injection, resulted in outgrowth of representative lesions (Boretto et al., 2019). Recently, Esfandiari et al. (2021) also developed organoids from endometriotic biopsies and showed that methylation patterns from the primary tissue were robustly recapitulated. Now, to fully recapitulate the inflammatory character and immunological dysregulation of endometriosis, epithelial organoid cultures should be enriched with stromal cells that also play a role in endometriosis, and further with endothelial and immune cells, while provided with a scaffold that allows for angiogenesis, neurogenesis and immune cell influx. Such efforts should also be applied to adenomyosis, for which epithelial organoids have not yet been described. Before, a co-culture model of adenomyotic epithelial cells, stromal cells and myocytes has been reported (Mehasseb et al., 2010).

### Endometrial Hyperplasia and Cancer

Endometrial cancer (EC) is the second most common tumor of the FRT and its incidence is rising incessantly in industrialized countries, subjecting women to cancer- and therapy-related risks (Bray et al., 2018; Zhang et al., 2019b). EC is a heterogenic constellation of diseases for which etiopathogenesis has historically been dichotomized into two groups based on clinico-histological characteristics (Bokhman, 1983). Type I tumors were postulated to be estrogen-mediated, well-to-moderately differentiated endometrioid lesions on a background of juxtaposed hyperplasia in younger women. Type II EC referred to poorly differentiated tumors of endometrioid or non-endometrioid histology arising in a milieu of endometrial atrophy and were claimed to be estrogen-independent. There is also an important role for hereditary syndromes such as Lynch syndrome, that can result in endometrial cancers of both categories as well as a multitude of extra-uterine malignancies (Lynch et al., 2015). Sequencing efforts by The Cancer Genome Atlas (TCGA) Research Network later fine-tuned the knowledge of EC-related mutations and allowed for a more precise (i.e., resulting in superior distinction of low- versus high-risk EC) molecular classification that is outside the scope of this review (Cancer Genome Atlas Research Network, 2013).

To date, the identity of the primordially affected cells in humans is still unknown. Syed et al. (2020) advanced AXIN2+ (stem) cells in mice to be EC-initiating cells upon oncogenic transformation. Before, most insights were drawn from patient EC-derived cell lines such as Ishikawa and ECC-1 (well-differentiated), RL95-2, HEC1A and HEC1B (moderately differentiated), and KLE and AN3CA (metastatic, poorly differentiated) (Van Nyen et al., 2018). Genomic profiling showed their comparative and temporal stability with respect to copy number aberrations and EC-associated point mutations, but intra-tumor heterogeneity was lost in cell lines. Mouse xenograft models, starting from these cell lines or primary tumors (105–107) showed fair engraftment rates of about 60% although aggressive subtypes are more efficient in growing out (Wang et al., 2010; Cabrera et al., 2012; Korch et al., 2012). The xenografts showed 90% genetic similarity with the tumor (Depreeuw et al., 2015), displaying only low numbers of newly acquired SCNAs, but genetic drift was still observed (Ben-David et al., 2017). Further limitations of EC PDX are the inability to replicate full intra-tumor heterogeneity, to mimic the tumor micro-environment as mouse stroma gradually replaces the human stroma present in the transplanted tumor, the absence of immunomodulatory responses and difficulties to correctly simulate patient drug-responses (Depreeuw et al., 2015). With respect to representativeness, another step in this direction was taken by implementing 3D culturing techniques, as exemplified by spheroid cultures which were instrumental to uncover altered metabolism, polarity and drug susceptibility (Chitcholtan et al., 2012, 2013). Spheroid constructs have been applied to study carcinogenesis, either in isolation or as element in a co-culture or explant model (Hashimoto et al., 2017; Goad et al., 2018; Al-Juboori et al., 2019). To provide a more accurate rendering of EC, research groups embarked on establishing organoids from EC tumor samples (Dasari et al., 2017; Girda et al., 2017; Pauli et al., 2017; Turco et al., 2017; Boretto et al., 2019; Table 1 and Supplementary Table 2). Organoid development was achieved, reliant on the typical factors such as RSPO1, Noggin, EGF, FGF2, FGF10, A83-01, NAC, and NAM, further promoted by addition of insulin-like growth factor 1 (IGF1), hepatocyte growth factor (HGF) and lipids. Importantly, it was necessary to lower p38i concentration to favor organoid growth from tumor cells above growth from healthy cells, also often present in the original biopsy. Interleukin-6 (IL6) and (non-)essential amino acids were also tested, but proved less important (Boretto et al., 2019). In addition to IHC and gene-expression characterization of the tumor organoids for endometrium/EC markers like estrogen receptor-α (ERα), progesterone receptor (PR), CK AE1/AE3, CK7, CK20, mucin 1 (MUC1), SRY-Box transcription factor 17 (SOX17), cluster of differentiation 10 (CD10), CD44 and/or aldehyde dehydrogenase 1 (ALDH1), two studies also defined and validated genomics and transcriptomics of the EC-derived organoids (Pauli et al., 2017; Boretto et al., 2019). Based on recapitulated mutations in AT-rich interaction domain 1A (*ARID1A*), β-Catenin (*CTNNB1*), F-box and WD repeat domain containing protein 7 (*FBXW7*), human epidermal growth factor receptor 2 (*HER2*), Polymerase-ε exonuclease domain (*POLE*) and phosphatase and tensin homolog (*PTEN*), organoids were tested for sensitivity to classic chemotherapeutics (5-fluorouracil, carboplatin, paclitaxel and doxorubicin), phosphoinositide 3-kinase (PI3K) inhibitors (apitolisib, buparlisib), inhibitors of mammalian target of rapamycin (mTOR) (everolimus), and histone deacetylase (HDAC) inhibitors (vorinostat, belinostat). Our group showed the organoids’ ability to reproduce the phenotype of the spectrum of endometrial states, i.e., from healthy, over simple and complex hyperplasia with and without atypia, to cancerous endometrium, and to recapitulate mutations of Lynch syndrome patients (Boretto et al., 2019). Now that the necessary backbone is provided, next-generation organoid-based models can be developed including co-culture systems, which will lend themselves to extensive, or rather focused, drug testing and cutting-edge gene-editing exploration.

## Fallopian Tubes

**FIGURE 5:**
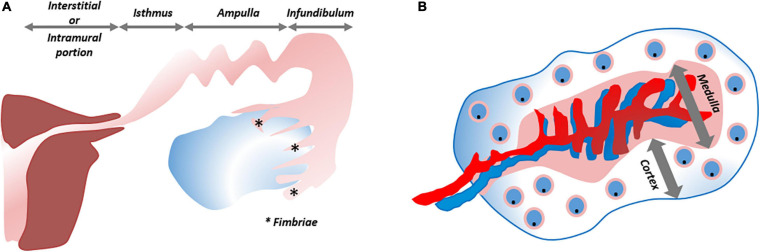
Schematic overview of fallopian tube and ovary. **(A)** The fallopian tube consists of four zones with distinct histological architecture, from distal to proximal: infundibulum with its fimbriae (*), ampulla, isthmus and interstitial/intramural portion. **(B)** The ovary is divided into a cortical region in which the oocytes/follicles develop and the medulla that contains the larger blood vessels. ***Refers to “Fimbriae”.

The FT (or oviducts), functioning as relay between uterus and ovaries, consist of four zones with distinct histological architecture, i.e., the fimbriated and funneled infundibulum, the tortuous ampulla, the muscular isthmus and the circular and myometrialized interstitial/intramural portions (Figure 5A). In order to provide an appropriate environment for gamete conditioning, fertilization and ovum nutrition, and to allow proper transit of the zygote, secretory, ciliary and muscular functions of the different parts are aligned (Jarboe, 2014).

Since the first *in vitro* culturing of oviductal cells (McComb et al., 1986), several tactics have been applied to overcome their rapid senescence, loss of polarization, fibroblast overgrowth and deciliation/dedifferentiation (Henriksen et al., 1990; Ando et al., 2000; Levanon et al., 2010; Karst et al., 2011). Karst et al. (2011) immortalized 2D-grown oviductal cells, and showed their tumorigenicity upon injection in immunocompromised mice. Using a transwell approach, the same group also cultured non-immortalized fimbrial FT epithelial cells at air/liquid interface, thereby replicating typical structural, proteomic and secretomic features, and providing insight into DNA damage repair kinetics (Levanon et al., 2010). To surmount the yet limited propagation of this system, and the failure to simulate the oviducts’ tubular folded architecture, further advanced 3D constructs were generated by placing minced mouse or baboon oviducts in alginate matrix, exposed to a precisely defined cocktail of hormones and growth factors (King et al., 2011). The immersed cells expressed the FT markers oviductin (OVGP1), paired box 8 (PAX8), E-cadherin, CK8 and acetylated tubulin (in cilia), and phenocopied the normal cell proliferation rate of the healthy donor. The main drawback was the limited culturing capacity (only for 7 days). The identification of label-retaining, putative stem cells in the distal oviduct of mice formed the impetus toward 3D organoid modeling using Matrigel (Wang et al., 2012). Grown under serum-free conditions, requiring only FGF2 and EGF, the organoid cells remained in an undifferentiated, slow-proliferative state for at least 10 weeks. Adding serum nudged cells to differentiate into various Müllerian derivatives, as exemplified by different expression patterns of ERα, PR, CD44, and progestagen associated endometrial protein (PAEP), and by the formation of hollow tubal structures through budding-out of the differentiating organoids. Kessler et al. (2015) were the first to develop organoids from human epithelial (epithelial cell adhesion molecule (EpCAM+) FT cells (Table 1 and Supplementary Table 4). Wnt potentiation was indispensable for organoid propagation, and the Wnt-boosting LGR6 emerged as potential FT stem cell marker. Interestingly, *LGR6* expression was also found upregulated in endometriotic organoids when compared to healthy endometrium organoids (Boretto et al., 2019). Optimal expansion of FT organoids required WNT3A, RSPO1, EGF, FGF10, TGF-βr kinase inhibitor (SB431542) and Noggin. Both secretory and ciliated cells were present, giving credence to the possible existence of a common bipotent stem cell. Notch inhibition by use of a γ-secretase inhibitor propelled ciliogenesis (Kessler et al., 2015), as later also found in endometrial organoids (Haider et al., 2019). It was also demonstrated that the fimbrial ends of the oviducts possess the highest organoid-forming capacity, both in mouse (Xie et al., 2018) and human (Rose et al., 2020), and that human fimbrial ALDH+ cells replicate with higher frequency and form larger structures (Rose et al., 2020). Co-culturing of FT epithelial cells with FT stromal cells and endothelial (HUVEC) cells formed more complex organoid structures (Chang et al., 2020). FT epithelial cells could also be derived from induced pluripotent stem cells (iPSC) in which first an intermediate mesoderm state was induced, subsequently differentiated toward a FT phenotype *via* suppletion of WNT4 (or WNT3A), follistatin, E2 and P4 (Yucer et al., 2017).

The FT organoids were applied to explore the origin of ovarian cancer (OC) and to model infections with specific pathogens. Kopper et al. (2019) defined the optimal culture conditions to obtain organoids from both healthy FT and ovarian surface epithelium (OSE), alongside organoids from a broad spectrum of OC subtypes including high-risk patients with germline mutations in breast cancer types 1 and 2 susceptibility genes (BRCA1/2), thus allowing to study how their (unaffected) FT-derived organoids relates to OC. Organoid formation from FT, OSE, and OC required equal amounts of RSPO1, Noggin, NAC, NAM, A83-01, and ROCKi. Organoid derivation from healthy FT as compared to healthy OSE asked for less Wnt, but similar EGF levels. Maintenance of OSE organoids additionally needed forskolin, hydrocortisone, E2 and the tyrosine kinase activator heregulin-β1 in amounts equivalent to OC organoids. Kopper et al. (2019) were able to further optimize OC organoid formation efficiency by reducting of EGF and adding FGF10. Moreover, it was observed that only a subset of OC benefited from Wnt supplementation, moreover added at lower concentrations than for organoid derivation from FT and OSE. Hoffmann et al. (2020) knocked down p53, PTEN and retinoblastoma (RB) tumor suppressor genes in FT organoids resulting in genomic instability, reduced apicobasal polarity, and larger and polymorphic nuclei compared to healthy controls. The modified organoids showed similarity to organoids derived from high-grade serous OC (HGSOC). In addition to sharing prominent morphological characteristics (such as nuclear atypia, increased DNA damage and altered epithelial organization) and transcriptomic traits (such as congruent upregulation of proto-oncogenes and downregulation of Wnt signaling), the medium optimized for HGSOC-derived organoids also promoted stemness in the modified FT organoids. Head-to-head comparisons between genetically rewired mouse FT and ovarian organoids supported that HGSOC can be derived from both cell populations, which may underlie HGSOC clinical heterogeneity, evidenced by differences in transcriptome, tumor kinetics and drug responses (Zhang et al., 2019a; Lõhmussaar et al., 2020). It has been proposed before that fimbrial ends of the FT form an equally important site of ovarian tumorigenesis (Dubeau, 1999; Karnezis et al., 2017). Thus, the FT organoids platform will allow researchers to delve deeper into the minimal (epi-)genetic requirements for oviductal tumorigenesis, using, amongst others, modern gene-editing techniques.

Acute and chronic salpingeal infections may cause progressive pelvic inflammatory disease and subsequent ectopic pregnancies and/or subfertility. With the emergence of multidrug-resistant sexually transmitted diseases, it is of paramount importance to have preclinical models to study host-microbe interplay, query treatments for infections, and develop regenerative therapies in case of surgical resections (Harris et al., 2018). Infection with *Chlamydia trachomatis* has recently been modeled in human FT organoids as well as in mouse endometrial organoids (Kessler et al., 2019; Bishop et al., 2020). Acute infection triggered a sustained response of inflammation and homeostatic repair. Infected cells containing bacterial inclusions extruded into the organoid lumen, after which adjacent cells compensatorily repleted their inlet. Owing to organoid longevity, also chronic chlamydiosis, known for its subclinical presentation, could be studied. Chronic infection of the organoids could model trace effects of infection, convalescence and reinfection, resulting in significantly increased organoid-forming capacity and increased EpCAM expression, even after successful treatment and curation of the chronic infection. A shift toward a less differentiated, secretory phenotype and epigenetic rewiring akin to aging are features reminiscent of OC.

## Ovaries

The ovaries, consisting of a central medulla, peripheral cortex and overlying serosa, serve two main purposes: steroidogenesis and iterative oocyte maturation with subsequent transmission into the FT. The cortical region houses the developing oocytes/follicles (White et al., 2012; Figure 5B). It has been speculated many times that oogonial stem cells (“cortical reserve”) may exist, but this hypothesis remains controversial and heavily debated. Cells expressing extracellular DEAD-box polypeptide 4 (ecDDX4) have been advanced as stem cell candidates, but recent single-cell omics identified these ecDDX4+ cells as perivascular cells (Fan et al., 2019; Wagner et al., 2020). To date, the Zuckerman axiom that a fixed number of oocytes is present and available throughout a woman’s lifetime still stands (Zuckerman, 1951). Regarding the cortex-bordering OSE, organoid studies, as described above, may eventually lead to the identification of the OSE stem cells, which may also lie at the origin of epithelial ovarian cancer (EOC). With more than half of affected women succumbing to this disease, EOC is considered the most lethal gynecological cancer. This high death-to-incidence ratio is attributable to the fact that the majority of cases are diagnosed in advanced stages of disease, and due to rapid recurrences (Prat and FIGO Committee on Gynecologic Oncology, 2014; Lheureux et al., 2019). EOC is more than a single entity and comprises serous, mucinous, endometrioid and clear cell histological signatures. Exact origin and downstream pathobiology remain debated. As previously mentioned, both FT epithelium and OSE have been proposed and validated as originating tissues for EOC (Dubeau, 1999; Jarboe, 2014; Coscia et al., 2016; Karnezis et al., 2017; Zhang et al., 2019a; Hoffmann et al., 2020; Lõhmussaar et al., 2020). One-fifth of the patients are genetically predisposed (Walsh et al., 2011; Toss et al., 2015), and advancements in genetic testing, biomarker discovery and preclinical models are enabling the application of personalized therapies. Cell lines of EOC, used as preclinical models, suffer from genetic drift, cross-contamination and misidentification (Sandberg and Ernberg, 2005; Korch et al., 2012; Domcke et al., 2013). In particular, SK-OV-3 and A2780, the two most utilized cell lines to emulate HGSOC, lack its typical *TP53* mutation and distinctive somatic copy number alterations (SCNA). Instead, they harbor mutations typical for other histotypes (e.g., *ARID1A* and *PTEN*) (148). Given this incongruity as well as poor translatability with regards to clinical response, these cell lines are not highly apt as preclinical model of HGSOC (Matsuo et al., 2010). Xenografts grown from the cell lines face the same dire fate (Bobbs et al., 2015). Transgenic mouse models, although allowing to query early events in ovarian tumorigenesis, insufficiently recapitulate the full genomic landscape of HGSOC (Bobbs et al., 2015). Patient-derived xenografts in immunodeficient mice reproduce relevant HGSOC complications such as tumor invasion, expansion and metastasis, and retain histologic and genomic characteristics (at least at early passage), but lack the immune component (Dobbin et al., 2014; Weroha et al., 2014; Bobbs et al., 2015). In order to better reproduce tumor complexity, *in vitro* 3D spheroid models have been developed. Differentially expressed genes, altered tumor kinetics and more translatable drug responses have been noted using spheroids as compared to 2D cell lines (Zietarska et al., 2007; Raghavan et al., 2015). Immune and stromal components were added to scrutinize cancer stem cell pathways and cellular interactions (Raghavan et al., 2019). Spheroids were also used to study progression from normal OSE to preinvasive phenotypes (e.g., by tracking depolarization, disorganized stratification, overexpression of cancer markers and ultimately degradation of the subjacent basement membrane) (Kwong et al., 2009). Studying these first steps from normal to invasive phenotype required OSE cells to be cultured in Matrigel-coated wells while suspended in a serum-containing medium supplemented with 2% Matrigel and continuously exposed to tumor necrosis factor α (TNF-α). The importance of TNF-α supports the association of tumorigenesis with chronic inflammation. The spheroids arose by aggregation, not by self-organization, and could not be maintained beyond 40 days. Nevertheless, the short-term 3D spheroid cultures were more representative than 2D setups when assessing OC drug sensitivities (Jabs et al., 2017).

Significant progress in OC modeling was generated by the development of organoids from OC as mentioned above. Organoid lines were established from a wide variety of OC, ranging from borderline tumors to invasive OC of various grades, stages and histologies (Kopper et al., 2019; Table 1 and Supplementary Table 4). A preceding study showed that organoid establishment was feasible without forskolin, hydrocortisone, E2, heregulin-β1 and ROCKi but required FGF2, PGE2 and p38i, although organoid maintenance was only short-term and the study focused mainly on HGSOC (Hill et al., 2018). Subsequent studies emphasized the importance of heregulin-β1 (neuregulin-1) and low WNT environment for long-term EOC organoid expansion (Hoffmann et al., 2020; Maenhoudt et al., 2020). The OC-derived organoids mirrored the (epi-)genomic and transcriptomic landscape of their native tissue and exemplified tumor heterogeneity at different sites of metastatic disease (Kopper et al., 2019). Organoid gene transcript clustering provided arguments for the hypothesis that borderline tumor may transition into OC. Organoids were amenable to gene-editing, xenografting and drug-sensitivity profiling (Hill et al., 2018; Kopper et al., 2019; Maru et al., 2019b; Phan et al., 2019; Chen et al., 2020; Hoffmann et al., 2020; Lõhmussaar et al., 2020; Maenhoudt et al., 2020). Hill et al. (2018) functionally studied HGSOC-specific homologous recombination deficiency (HRD) and replication fork instability, and observed that functional assays (i.e., drug-testing) systematically outperformed the data obtained from mutational profiles alone, moreover in keeping with the parallel clinical reality. Further developments were achieved to circumvent the possible interference of the gel-based scaffold with drug diffusion (using resuspended organoids) and to apply the organoid platform in high-throughput settings (Maru et al., 2019b; Phan et al., 2019). Drug sensitivity was linked to well-recapitulated individual (epi-)genomic and transcriptomic profiles (Phan et al., 2019; Chen et al., 2020; de Witte et al., 2020; Nanki et al., 2020; Zhang et al., 2020). To a further extent, the organoids reliably simulated intra- and inter-patient drug response heterogeneity and were able to predict useful therapeutic agents in the majority of cases, longitudinally compared to patient-wide clinical outcomes albeit retrospectively (de Witte et al., 2020). These findings put us at the brink of a new era in which the predictive value of OC-derived organoids will be prospectively tested in clinical trials. The prospect of more complex organoid models also encompassing immune, stromal and/or vascular cells, raises the hope to in the future test clinically relevant drugs that tackle neo-angiogenesis and tumor immunology. Indeed, short-term 3D spheroid co-cultures of HGSOC cells with immune cells have shown sensitivity to immune checkpoint inhibitors (Wan et al., 2020).

## Concluding Remarks

Over the past decades, we have witnessed an important paradigm shift in preclinical modeling of healthy and diseased tissues. With respect to the organs of the female reproductive system concentrated in and near the pelvis, organoids have gradually assumed a pivotal position in research. Considering their efficient establishment and propagation, their amenability to state-of-the-art techniques (such as gene-editing and single-cell omics), their *in vivo* transplantability and their positive translational power, this promising technology has spurred an invaluable amount of *in vitro* and *in vivo* realizations. Organoids provide an unprecedented opportunity to practice personalized medicine. The most important hurdle that lies ahead is to further enrich the established organoid culturing systems, by adding stromal, vascular and immune components to still better mimic real-life conditions. Rightfully wielding this momentum by means of parallel clinical trials (Vlachogiannis et al., 2018; Ooft et al., 2019) will now be of paramount importance to further close the gap between bench and bedside.

## Author Contributions

RH devised the idea, performed the literature search, and wrote the manuscript. ZJ performed a simultaneous and focused organoid-specific literature search. RH and ZJ independently constructed the tables. RH designed and adapted the figures. HV amended and co-wrote the manuscript. DT and HV provided funding and essential insights to produce this manuscript. All authors critically appraised the manuscript, which was finalized by RH.

## Conflict of Interest

The authors declare that the research was conducted in the absence of any commercial or financial relationships that could be construed as a potential conflict of interest.

## Supplementary Material

Supplementary Table 1 Recruitment details and medium compositions of human vulvar, vaginal and cervical organoid studies.

Supplementary Table 2 Recruitment details and medium compositions of human endometrial organoid studies.

Supplementary Table 3 Recruitment details and medium compositions of pregnancy-related organoid studies.

Supplementary Table 4 Recruitment details and medium compositions of human ovarian organoid studies.
