# Supplementary material for: Organoids of the Female Reproductive Tract: Innovative Tools to Study Desired to Unwelcome Processes
Source: Front Cell Dev Biol. 2021 Apr 20;9:661472. doi: 10.3389/fcell.2021.661472 (PMC8093793; doi:10.3389/fcell.2021.661472)
Supplement: Supplementary Table 1 — Recruitment details and medium compositions of human vulvar, vaginal and cervical organoid studies. [file Table_1.docx]

**Supplementary Tables**

**Organoids of the female reproductive tract:
innovative tools to study desired to unwelcome processes**

Ruben Heremans, Ziga Jan, Dirk Timmerman, Hugo Vankelecom

| **Supplementary Table 1.** Recruitment details and medium compositions of human vulvar, vaginal and cervical organoid studies | | | | | | | | | | | | | | | | |
| --- | --- | --- | --- | --- | --- | --- | --- | --- | --- | --- | --- | --- | --- | --- | --- | --- |
| **Author, Year** | **Subjects** | **Medium name** | **EGF Pathway** | **WNT Pathway** | | | **BMP**  **inhibition** | **FGF Pathway** | **HGF Pathway** | **Small molecules** | | | **Hormones** | | **Other relevant ingredients** | |
|  |  |  | **EGF (ng/mL)** | **Wnt3a^¶^** | **R-SPO-1^¶^** | **CHIR 99021**  **(µM)** | **Noggin^¶^** | **FGF-2/-7/**  **FGF-10**  **(ng/mL)** | **HGF**  **(ng/mL)** | **A83-01/**  **SB 43152**  **(nM)** | **NAM (mM)** | **Y-27632**  **(ROCKi)**  **(µM)** | **E2**  **(nM)** | **Other hormones/**  **small molecules** | | **Fetal calf serum**  **(%)** |
| Vulva | | | | | | | | | | | | | | | | |
|  |  |  |  |  |  |  |  |  |  |  |  |  |  |  | |  |
| Vagina | | | | | | | | | | | | | | | | |
|  |  |  |  |  |  |  |  |  |  |  |  |  |  |  | |  |
| Cervix: ectocervix, endocervix - cervical cancer | | | | | | | | | | | | | | | | |
| Chumduri et al., 2017  *and*  Chumduri et al.,  2021 | Healthy ectocervix,  N=NA | Wnt deficient | 10 |  |  |  | 100 | FGF-10:  100 |  |  | 10 | 10 |  | TGF-β RI kinase Inhibitor IV: 2 µM Hydrocortisone: 500 ng/mL;  Forskolin:10 µM  NAC: 1.25mM; | |  |
|  | Healthy endocervix, N=NA | Wnt proficient | 10 | 25% | 25% |  | 100 | FGF-10:  100 |  |  | 10 | 10 |  | TGF-β RI kinase Inhibitor IV: 2 µM  NAC: 1.25 mM | |  |
| Maru et al., 2019b | Clear cell cervical cancer,  N=1 |  | 50 |  | 250 |  | 100 |  |  |  |  | 10 |  | Jagged-1:  1 μM | |  |
| Maru et al., 2020 | Healthy cervical biopsies,  N=4 |  | 50 |  | 250 |  | 100 |  |  |  |  | 10 |  | Jagged-1:  1 μM | |  |

**¶ Provided in ng/mL or % Conditioned medium (CM)**
