# Supplementary material for: Organoids of the Female Reproductive Tract: Innovative Tools to Study Desired to Unwelcome Processes
Source: Front Cell Dev Biol. 2021 Apr 20;9:661472. doi: 10.3389/fcell.2021.661472 (PMC8093793; doi:10.3389/fcell.2021.661472)
Supplement: Supplementary Table 2 — Recruitment details and medium compositions of human endometrial organoid studies. [file Table_2.docx]

| **Supplementary Table 2.** Recruitment details and medium compositions of human endometrial organoid studies | | | | | | | | | | | | | | | | |
| --- | --- | --- | --- | --- | --- | --- | --- | --- | --- | --- | --- | --- | --- | --- | --- | --- |
| **Author, Year** | **Subjects** | **Medium name** | **EGF Pathway** | **WNT Pathway** | | **BMP**  **inhibition** | **FGF Pathway** | **HGF Pathway** | **Small molecules** | | | **p38 MAPK-Inhibition** | **Hormones** | | **Other relevant ingredients** | |
|  |  |  | **EGF (ng/mL)** | **Wnt3a^¶^** | **R-SPO-1^¶^** | **Noggin^¶^** | **FGF-2/-7/**  **FGF-10**  **(ng/mL)** | **HGF**  **(ng/mL)** | **A83-01**  **or**  **SB 43152**  **(nM)** | **NAM (mM)** | **Y-27632**  **(µM)** | **SB 202190**  **(µM)** | **E2**  **(nM)** | **Other hormones or small molecules** | | **Fetal calf serum**  **(%)** |
| **Endometrium: atrophic, proliferative, secretory - Endometrial hyperplasia - Endometriosis: eutopic, ectopic - Endometrioid endometrial cancer (EEC) - other EC** | | | | | | | | | | | | | | | | |
| Turco et al., 2017;  Fitzgerald et al. 2019;  Luddi et al., 2020 | Atrophy, N=1;  Proliferative, N=3;  Secretory, N=11;  EEC, N=3 | ExM | 50 |  | 500 | 100 | FGF-10:  100 | 50 | A83-01:  500 | 10 | 10 |  | 10**^§^** | NAC: 1.25 mM  P4**^§^**^:^ 1 µM,  Prolactin**^§^**: 20 ng/mL,  hCG**^§^**: 1 µg/mL  hPL**^§^**: 20 ng/mL  cAMP**^§^**: 1 µM; | | 10^†^ |
| Boretto et al., 2017;  Hennes et al., 2019 | All phases, N=N/A | Human organoid culture medium | 50 | 200 | 200 | 100 | FGF-10:  50 |  | A83-01:  500 | 1 | 9* | 10 | 2 | Insulin: 1 %;  NAC: 1.25 mM  P4**^§^**: 50 ng/mL | | 10^†^ |
| Dasari et al., 2017 | EEC, N=N/A |  | 20 |  |  |  | FGF-2:  10 |  |  |  |  |  |  | Insulin: 50 µg/mL  BSA: 0.4% | |  |
| Girda et al., 2017 | EEC, N=15 |  |  |  |  |  |  |  | A83-01:  500 | 10 | 10 | 3 | 1 |  | |  |
| Pauli et al., 2017 | EEC, N=1  Other EC, N=1 | CHM | 50 |  | 25% | 50% | FGF-2:  1  FGF-10:  20 |  | A83-01:  500 | 10 | 10 | 10 |  | NAC: 1.25 mM  PGE2: 1 µM | | 10^†^ |
| Boretto et al., 2019  *and*  Esfandiari et al, 2020 | Healthy, N=15/9;  Endometriosis, N=21/16;  Hyperplasia, N=11; | Standard | 50 |  | 10% | 100 | FGF-2:  2  FGF-10:  10 |  | A83-01:  500 | 2 | 10* | 10 | 1 | Insulin: 1%;  NAC: 1.25 mM | | 10^†^ |
|  | EEC, N=11;  Other EC, N=2 | Optimized | 50 |  | 5% | 100 |  | 20 | A83-01:  250 | 5 | 10* | 0.1 | 10 | IGF-1: 40 ng/mL  NAC: 1.25 mM  Chemically defined lipid concentrate: 1% | | 10^†^ |
| Maru et al., 2019a | EEC,  N=5  Other EC,  N=1 |  | 50 |  | 250 | 100 |  |  |  |  | 10 |  |  | Jagged-1:  1 μM | | 10^‡^ |
| Cochrane et al., 2020 | Secretory,  N=3 | ExM | 10 | 25% | 25% | 100 | FGF-10:  100 |  | SB 431542:  500 | 1 | 9 |  |  |  | | 5 |

**¶ Provided in ng/mL or % Conditioned medium (CM)
† during sample dissection/digestion
‡ during sample seeding
§ for differentiation*** **only for formation or dissociation at passaging**
