# Supplementary material for: Organoids of the Female Reproductive Tract: Innovative Tools to Study Desired to Unwelcome Processes
Source: Front Cell Dev Biol. 2021 Apr 20;9:661472. doi: 10.3389/fcell.2021.661472 (PMC8093793; doi:10.3389/fcell.2021.661472)
Supplement: Supplementary Table 3 — Recruitment details and medium compositions of pregnancy-related organoid studies. [file Table_3.docx]

| **Supplementary Table 3.** Recruitment details and medium compositions of pregnancy-related organoid studies | | | | | | | | | | | | | | | | |
| --- | --- | --- | --- | --- | --- | --- | --- | --- | --- | --- | --- | --- | --- | --- | --- | --- |
| **Author, Year** | **Subjects** | **Medium name** | **EGF Pathway** | **WNT Pathway** | | | **BMP**  **inhibition** | **FGF Pathway** | **HGF Pathway** | **Small molecules** | | | **Hormones** | | **Other relevant ingredients** | |
|  |  |  | **EGF (ng/mL)** | **Wnt3a^¶^** | **R-SPO-1^¶^** | **CHIR 99021**  **(µM)** | **Noggin^¶^** | **FGF-2/-7/**  **FGF-10**  **(ng/mL)** | **HGF**  **(ng/mL)** | **A83-01/**  **SB 43152**  **(nM)** | **NAM (mM)** | **Y-27632**  **(ROCKi)**  **(µM)** | **E2**  **(nM)** | **Other hormones/**  **small molecules** | | **Fetal calf serum**  **(%)** |
| Decidual endometrium | | | | | | | | | | | | | | | | |
| Turco et al., 2017 | Decidua,  N=25 | ExM | 50 |  | 500 |  | 100 | FGF-2:  100 | 50 | A83-01:  500 | 10 | 10 | 10**^§^** | P4**^§^**^:^ 1µg/mL  Prolactin**^§^**: 20ng/mL,  hCG**^§^**: 1µg/mL  hPL**^§^**: 20ng/mL | | 10 |
| Haider et al., 2019 | Decidua,  N=24 | ERNAC | 100 |  | 100 | 3 | 100 |  |  | A83-01:  10 000 |  |  |  | PGE2: 2.5 μM | |  |
| Trophoblast: first trimester placenta, term placenta | | | | | | | | | | | | | | | | |
| Haider et al., 2018 | First trimester placenta,  N=16 | a-TOM | 100 |  | 100 | 3 | 100 |  | 50 | A83-01:  1000 |  |  |  | PGE: 2.5 μM | |  |
| Turco et al., 2018 | First trimester placenta,  N=22 | TOM | 50 |  | 80 | 1.5 |  | FGF-2:  100 | 50 | A83-01:  500 |  | 2 |  | PGE2: 2.5 μM  NAC: 1.25 mM | | 20**^†^** |
| Marinic et al., 2020 | Term placenta,  N=6 | ExM | 50 |  | 500 |  | 100 | FGF-10:  100 | 50 | A83-01:  500 | 10 |  |  | NAC: 1.25 mM | |  |

**¶ Provided in ng/mL or % Conditioned medium (CM)
† during sample dissection/digestion
‡ during sample seeding
§ for differentiation**
