# Supplementary material for: Organoids of the Female Reproductive Tract: Innovative Tools to Study Desired to Unwelcome Processes
Source: Front Cell Dev Biol. 2021 Apr 20;9:661472. doi: 10.3389/fcell.2021.661472 (PMC8093793; doi:10.3389/fcell.2021.661472)
Supplement: Supplementary Table 4 — Recruitment details and medium compositions of human ovarian organoid studies. [file Table_4.docx]

| **Supplementary Table 4.** Recruitment details and medium compositions of human fallopian tube and ovarian organoid studies | | | | | | | | | | | | | | | | | | |
| --- | --- | --- | --- | --- | --- | --- | --- | --- | --- | --- | --- | --- | --- | --- | --- | --- | --- | --- |
| **Author, Year** | **Subjects** | **Medium name** | **EGF**  **Pathway** | | **WNT**  **Pathway** | | **BMP**  **inhibition** | **FGF Pathway** | **HGF Pathway** | **Small molecules** | | | **p38 MAPK-Inhibition** | **Hormones** | | | **Other relevant ingredients** | |
|  |  |  | **EGF (ng/mL)** | **NRG-1/**  **HRG-β1 (ng/mL)** | **Wnt3a^¶^** | **RSPO1^¶^** | **Noggin^¶^** | **FGF-2/-7/**  **FGF-10**  **(ng/mL)** | **HGF**  **(ng/mL)** | **A83-01/**  **SB 431542/**  **(nM)** | **NAM (mM)** | **Y-27632**  **(µM)** | **SB 202190**  **(µM)** | **E2**  **(nM)** | **Other hormones or small molecules** | | | **Fetal calf serum**  **(%)** |
| **Fallopian tube epithelium (FTE) - Ovarian surface epithelium (OSE) - benign ovarian lesions - ovarian borderline tumours (BOT) - low/high-grade serous ovarian cancer (L/HGSOC) - other epithelial ovarian cancers (EOC)** | | | | | | | | | | | | | | | | | | |
| Kessler et al., 2015  *and*  Kessler et al., 2019 | Healthy FT,  N=NA |  | 10 |  | 25% | 25% | 100 | FGF-2:  100 |  | SB 431542:  500 | 1 | 9 |  |  |  | | | 2-5 |
| Hill et al., 2018 | LGSOC,  N=1;  HGSOC,  N=21  Other EOC,  N=1 |  | 50 |  |  | 100 | 100 | FGF-2:  10  FGF-10:  10 |  | A83-01:  500 | 10 |  | 10 |  | PGE2: 1 μM  NAC: 1.25 mM | | |  |
| Kopper et al., 2019  *and*  de Witte et al,2020 | Healthy FT,  N=2;  BRCA_mut_ FT/OSE,  N=12/7 | FT | 12.5 |  | 25% | 10% | 1% |  |  | A83-01:  500 | 10 | 5 |  |  |  | | |  |
|  |  | OSE | 12.5 | 37.5 | 50% | 10% | 1% |  |  | A83-01:  500 | 10 | 5 |  | 100 | Hydrocortisone: 500 ng/mL;  Forskolin: 10 µM | | |  |
|  | BOT,  N=7;  LGSOC,  N=9  HGSOC,  N=17;  Other EOC,  N=7 | OC | 5 | 37.5 |  | 10% | 1% | FGF-2:  10 |  | A83-01:  500 | 10 | 5 |  | 100 | Hydrocortisone: 500 ng/mL;  Forskolin: 10 µM | | |  |
|  |  | OCwnt | 5 | 37.5 | 20% | 10% | 1% | FGF-2:  10 |  | A83-01:  500 | 10 | 5 |  | 100 | Hydrocortisone: 500 ng/mL;  Forskolin: 10 µM | | |  |
| Maru et al., 2019 | BOT,  N=5;  HGSOC,  N=4  Other EOC,  N=6 |  | 50 |  |  | 250 | 100 |  |  |  |  | 10 |  |  | Jagged-1  1 μM | | | 10^‡^ |
| Hoffmann et al,2020 | Fallopian tube,  N = NA;  HGSOC,  N=13 | Fallopian Tube Medium | 10 |  | 25% | 25% | 100 | FGF-2:  100 |  | A83-01:  500 | 1 | 9 |  |  |  | | | 10^‡^ |
|  |  | OC Minimal Medium | 10 |  |  |  |  |  |  | A83-01:  500 | 1 | 9 |  |  | BMP-2:  10 ng/mL | | | 10^‡^ |
| **Cont.** | | | | | | | | | | | | | | | | | | |
| **Supplementary Table 4.** Continued | | | | | | | | | | | | | | | | | | |
| **Author, Year** | **Subjects** | **Medium name** | **EGF**  **Pathway** | | **WNT**  **Pathway** | | **BMP**  **inhibition** | **FGF Pathway** | **HGF Pathway** | **Small molecule** | | | **p38 MAPK-Inhibition** | **Hormones** | | **Other relevant ingredients** | | |
|  |  |  | **EGF (ng/mL)** | **NRG-1/**  **HRG-β1 (ng/mL)** | **Wnt3a^¶^** | **RSPO1^¶^** | **Noggin^¶^** | **FGF-2/-7/**  **FGF-10**  **(ng/mL)** | **HGF**  **(ng/mL)** | **A83-01/**  **SB 43152/**  **(nM)** | **NAM (mM)** | **Y-27632**  **(µM)** | **SB 202190**  **(µM)** | **E2**  **(nM)** | **Other hormones or small molecules** | | | **Fetal calf serum**  **(%)** |
| Maenhoudt et al., 2020 | HGSOC,  N=27;  Other EOC,  N=5 | OCOM-4 | 50 | 50 |  | 50 | 100 |  | 10 | A83-01:  250 | 5 | 10 | 1 | 10 | IGF-1:  20 ng/mL | | | 10^†^ |
| Chen et al., 2020 | HGSOC,  N=6 | Complete medium | 5 | 5 |  | 10% | 100 | FGF-7:  5  FGF-10:  20 |  | A83-01:  500 | 1 | 5 | 0.5 |  | NAC: 1.25 mM | | |  |
| Nanki et al., 2020 | Benign lesions,  N=7:  BOT,  N=3;  HGSOC,  N=10;  Other EOC,  N=15; |  |  |  | 20% | 1000 | 100 | FGF-2:  50 |  | A83-01:  500 |  | 10 |  |  | NAC: 1 mM  IGF-1: 100 ng/mL  Leu15-Gastrin I: 10 nM | | |  |
| Zhang et al., 2020 | HGSOC,  N=9 |  | 50 | 50 | 0-25% | 25-50% | 100 | FGF-10:  10 |  | A83-01:  500 |  | 10 |  | 10***** | NAC: 1.25 mM  Forskolin: 10 µM | | |  |
| Rose et al., 2020 | Healthy FT |  | 10 |  | 25% | 10% | 100 | FGF-10:  100 |  | SB 431542: 2500 | 1 | 10 |  |  | Osteopontin: 100 U/mL | | |  |

**¶ Provided in ng/mL or % Conditioned medium (CM)
† during sample collection
‡ during sample seeding
* only for formation or dissociation at passaging**
